# 4-Nitro-2-phenoxy­aniline

**DOI:** 10.1107/S1600536810012237

**Published:** 2010-05-08

**Authors:** H. R. Manjunath, M. T. Shreenivasa, M. Mahendra, T. M. Mohan Kumar, B. E. Kumara Swamy, M. A. Sridhar

**Affiliations:** a Department of Studies in Physics, Manasagangotri, University of Mysore, Mysore 570 006, India; b Department of P. G. Studies and Research in Industrial Chemistry, Kuvempu University, Jnana Sahyadri, Shankaraghatta, Karnataka, India; c Chemistry Department, Amrita School of Engineering, Amrita Vishwa Vidyapeetham, Bangalore 560 035, India

## Abstract

In the title compound, C_12_H_10_N_2_O_3_, the oxygen atom bridging the two aromatic rings is in a synperiplanar (+*sp*) conformation. The dihedral angle between the aromatic rings is 71.40 (12)°. In the crystal, mol­ecules are linked by inter­molecular N—H⋯O hydrogen bonds.

## Related literature

For the pharmacological properties of nitro-2-phenoxy­aniline, see: Moore & Harrington (1974 ▶); Prasad *et al.* (2005 ▶). For the herbicidal applications of biphenyl ether derivatives, see: Yu *et al.*, (2008 ▶). For the applications of Schiff bases derived from aromatic amines, see: Singh *et al.* (1975 ▶); Cimerman *et al.* (2000 ▶). For their biological and pharmacological acitvity, see: Singh *et al.* (1975 ▶); Cimerman *et al.* (2000 ▶); Shah *et al.* (1992 ▶); Pandeya *et al.* (1999 ▶); More *et al.* (2001 ▶). For the preparation of 4-nitro-2-phenoxy­aniline, see: Shreenivasa *et al.* (2009 ▶). For a related structure, see: Naveen *et al.* (2006 ▶).
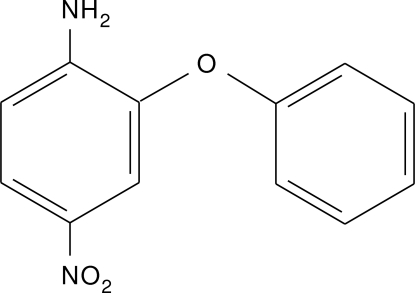


## Experimental

### 

#### Crystal data

C_12_H_10_N_2_O_3_
*M* *_r_* = 230.22
Monoclinic, 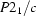

*a* = 10.4100 (12) Å
*b* = 15.6570 (18) Å
*c* = 6.9600 (17) Å
β = 103.406 (4)°
*V* = 1103.5 (3) Å^3^
*Z* = 4
Mo *K*α radiation
μ = 0.10 mm^−1^
*T* = 293 K
0.32 × 0.3 × 0.25 mm

#### Data collection

MacScience DIPLabo 32001 diffractometer
3336 measured reflections
1889 independent reflections
1498 reflections with *I* > 2σ(*I*)
*R*_int_ = 0.033

#### Refinement

*R*[*F* ^2^ > 2σ(*F* ^2^)] = 0.053
*wR*(*F* ^2^) = 0.167
*S* = 1.09
1889 reflections
154 parameters
H-atom parameters constrained
Δρ_max_ = 0.13 e Å^−3^
Δρ_min_ = −0.15 e Å^−3^

### 

Data collection: *XPRESS* (MacScience, 2002 ▶); cell refinement: *SCALEPACK* (Ot­win­owski & Minor, 1997 ▶); data reduction: *DENZO* (Otwinowski & Minor, 1997 ▶) and *SCALEPACK*; program(s) used to solve structure: *SHELXS7* (Sheldrick, 2008 ▶); program(s) used to refine structure: *SHELXL97* (Sheldrick, 2008 ▶); molecular graphics: *PLATON* (Spek, 2009 ▶) and *ORTEPII* (Johnson, 1976 ▶); software used to prepare material for publication: *SHELXL97*.

## Supplementary Material

Crystal structure: contains datablocks I, global. DOI: 10.1107/S1600536810012237/fj2288sup1.cif

Structure factors: contains datablocks I. DOI: 10.1107/S1600536810012237/fj2288Isup2.hkl

Additional supplementary materials:  crystallographic information; 3D view; checkCIF report

## Figures and Tables

**Table 1:** Hydrogen-bond geometry (Å, °)

*D*—H⋯*A*	*D*—H	H⋯*A*	*D*⋯*A*	*D*—H⋯*A*
N10—H10*A*⋯O9^i^	0.86	2.17	3.023 (3)	170

Symmetry code: (i) 
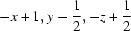
.

**Fig. 1.**
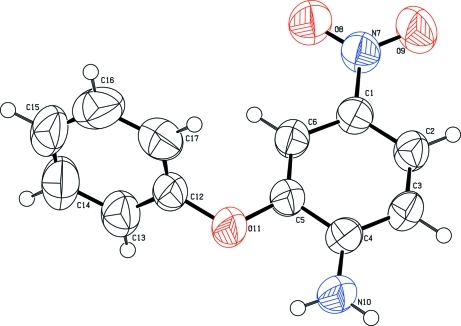
A view of (I), with 50% probability displacement ellipsoids.

**Fig. 2.**
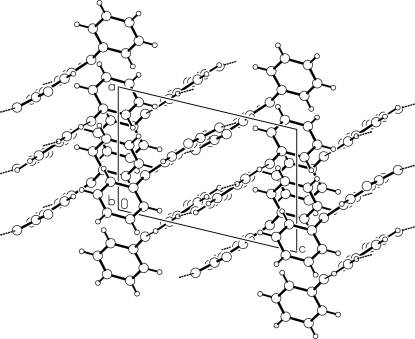
Packing diagram of the molecule viewed down the 'b' axis. The dotted lines represents the hydrogen bonds.

## supplementary crystallographic information

### Comment

The phenoxy anilines are versatile intermediates for synthesizing several pharmaceutical drugs i.e. Nimesulide, Ampxipine and Loxapine.  The Nitro-2-phenoxyaniline is an intermediate for the synthesis of Nimesulide and it was probably the first COX-2 selective non-steroidal anti inflammatory drug (NSAID) identified with this key pharmacological properties (Moore & Harrington, 1974; Prasad et al. 2005). It is a unique molecule with twin aromatic ring structure. The nitro-2-phenoxyaniline is a derivative of biphenyl ether. More generally, biphenyl ether derivatives have many biological, herbicidal (Yu et al., 2008) and organic chemistry applications. Schiff bases derived from aromatic amines have a wide variety of applications in many fields, viz., biological, inorganic and anlytical chemistry (Singh et al., 1975; Cimerman et al., 2000). They are known to exhibit potent antibacterial, anticonvulsant, anti-inflammatory (Shah et al. 1992), anticancer (Pandeya et al., 1999), anti-hypertensive and hypnotic (More  et al., 2001) activities. With this background, the title compound (I), was synthesized and we report its crystal structure here.

A perspective view of (I) is shown in Fig. 1. The two aromatic rings are not coplanar. This is confirmed by the dihedral angle value of 71.38 (12)° between two six-membered rings.  The oxygen atom connecting the two aromatic rings is in  syn-periplanar (sp) conformation as indicated by the torsion angle value of 13.0 (3)°. The nitro group lies in the plane of the aniline ring as indicated by the C2—C1—N7—O8 and C6—C1—N7—O9 torsion angles of -176.1 (2)° and -174.4 (2)°, respectively. These values are different from the values reported earlier (Naveen S. et al. 2006). The structure exhibits both inter and intramolecular N—H···O interaction. The intermolecular N10—H10A···O9 interaction has a length of 2.17Å and angle of 170° with symmetry codes 3/2-x,-1/2+y,1-z. The molecules exhibit layered stackings when viewd down the 'b' axis as shown in Fig. 2.

### Experimental

The 4-nitro-2-phenoxyaniline was prepared by condensation of *o*-chloronitrobenzene with phenol followed by acetylation and nitration (Shreenivasa *et al.*, 2009). The final product obtained was recrystallized using ethanol as a solvent. Colorless crystals were appeared after 4 days by slow evaporation.

### Refinement

H atoms were placed at idealized positions and allowed to ride on their parent atoms with C–H distances in the range 0.93–0.98 Å; *U*_iso_(H) = 1.2*U*_eq_(carrier atom) for all H atoms.

### Crystal data

**Table:**

C_12_H_10_N_2_O_3_	*F*(000) = 480
*M**_r_* = 230.22	*D*_x_ = 1.386 Mg m^−^^3^
Monoclinic, *P*2_1_/*c*	Mo *K*α radiation, λ = 0.71073 Å
Hall symbol: -P 2ybc	Cell parameters from 14613 reflections
*a* = 10.4100 (12) Å	θ = 2.4–32.5°
*b* = 15.6570 (18) Å	µ = 0.10 mm^−^^1^
*c* = 6.9600 (17) Å	*T* = 293 K
β = 103.406 (4)°	Block, colorless
*V* = 1103.5 (3) Å^3^	0.32 × 0.3 × 0.25 mm
*Z* = 4	

### Data collection

**Table:**

MacScience DIPLabo 32001 diffractometer	1498 reflections with *I* > 2σ(*I*)
Radiation source: fine-focus sealed tube	*R*_int_ = 0.033
graphite	θ_max_ = 25.0°, θ_min_ = 2.4°
Detector resolution: 10.0 pixels mm^-1^	*h* = −12→12
ω scan	*k* = −18→18
3336 measured reflections	*l* = −8→8
1889 independent reflections	

### Refinement

**Table:**

Refinement on *F*^2^	Primary atom site location: structure-invariant direct methods
Least-squares matrix: full	Secondary atom site location: difference Fourier map
*R*[*F*^2^ > 2σ(*F*^2^)] = 0.053	Hydrogen site location: inferred from neighbouring sites
*wR*(*F*^2^) = 0.167	H-atom parameters constrained
*S* = 1.09	*w* = 1/[σ^2^(*F*_o_^2^) + (0.0811*P*)^2^ + 0.2121*P*] where *P* = (*F*_o_^2^ + 2*F*_c_^2^)/3
1889 reflections	(Δ/σ)_max_ < 0.001
154 parameters	Δρ_max_ = 0.13 e Å^−^^3^
0 restraints	Δρ_min_ = −0.15 e Å^−^^3^

### Special details

**Table:**

Geometry. All esds (except the esd in the dihedral angle between two l.s. planes) are estimated using the full covariance matrix. The cell esds are taken into account individually in the estimation of esds in distances, angles and torsion angles; correlations between esds in cell parameters are only used when they are defined by crystal symmetry. An approximate (isotropic) treatment of cell esds is used for estimating esds involving l.s. planes.
Refinement. Refinement of *F*^2^ against ALL reflections. The weighted *R*-factor wR and goodness of fit *S* are based on *F*^2^, conventional *R*-factors *R* are based on *F*, with *F* set to zero for negative *F*^2^. The threshold expression of *F*^2^ > σ(*F*^2^) is used only for calculating *R*-factors(gt) etc. and is not relevant to the choice of reflections for refinement. *R*-factors based on *F*^2^ are statistically about twice as large as those based on *F*, and *R*- factors based on ALL data will be even larger.

### Fractional atomic coordinates and isotropic or equivalent isotropic displacement parameters (Å^2^)

**Table:**

	*x*	*y*	*z*	*U*_iso_*/*U*_eq_	
C1	0.4153 (2)	0.11783 (12)	0.1849 (3)	0.0575 (5)	
C2	0.4872 (2)	0.04830 (14)	0.2715 (3)	0.0624 (5)	
H2	0.5641	0.0561	0.3692	0.075*	
C3	0.4446 (2)	−0.03248 (13)	0.2129 (3)	0.0618 (5)	
H3	0.4920	−0.0794	0.2737	0.074*	
C4	0.3320 (2)	−0.04544 (12)	0.0647 (3)	0.0578 (5)	
C5	0.2615 (2)	0.02705 (13)	−0.0233 (3)	0.0637 (6)	
C6	0.3010 (2)	0.10777 (13)	0.0368 (3)	0.0643 (6)	
H6	0.2527	0.1550	−0.0200	0.077*	
N7	0.46086 (19)	0.20258 (12)	0.2446 (3)	0.0696 (5)	
O8	0.39280 (19)	0.26405 (10)	0.1748 (3)	0.0929 (6)	
O9	0.56703 (18)	0.21113 (11)	0.3643 (3)	0.0956 (6)	
N10	0.28775 (19)	−0.12433 (11)	0.0016 (3)	0.0742 (6)	
H10A	0.3300	−0.1689	0.0542	0.089*	
H10B	0.2174	−0.1298	−0.0909	0.089*	
O11	0.15146 (19)	0.00647 (10)	−0.1675 (3)	0.0991 (7)	
C12	0.0845 (2)	0.06917 (13)	−0.2929 (3)	0.0710 (6)	
C13	−0.0425 (2)	0.08508 (17)	−0.2880 (4)	0.0802 (7)	
H13	−0.0804	0.0568	−0.1974	0.096*	
C14	−0.1149 (3)	0.1424 (2)	−0.4153 (5)	0.0973 (9)	
H14	−0.2022	0.1528	−0.4110	0.117*	
C15	−0.0624 (4)	0.18388 (18)	−0.5465 (5)	0.1026 (10)	
H15	−0.1127	0.2234	−0.6318	0.123*	
C16	0.0659 (4)	0.16812 (19)	−0.5551 (4)	0.1079 (11)	
H16	0.1024	0.1967	−0.6469	0.129*	
C17	0.1418 (3)	0.10918 (18)	−0.4262 (5)	0.0934 (8)	
H17	0.2287	0.0976	−0.4309	0.112*	

### Atomic displacement parameters (Å^2^)

**Table:**

	*U*^11^	*U*^22^	*U*^33^	*U*^12^	*U*^13^	*U*^23^
C1	0.0635 (11)	0.0525 (10)	0.0538 (11)	0.0002 (9)	0.0079 (9)	−0.0054 (8)
C2	0.0669 (12)	0.0677 (13)	0.0474 (10)	0.0017 (10)	0.0026 (9)	0.0018 (9)
C3	0.0713 (13)	0.0586 (11)	0.0523 (11)	0.0093 (9)	0.0079 (9)	0.0073 (9)
C4	0.0647 (12)	0.0528 (11)	0.0553 (11)	0.0008 (9)	0.0125 (9)	0.0022 (8)
C5	0.0613 (12)	0.0559 (11)	0.0657 (12)	−0.0024 (9)	−0.0018 (10)	0.0025 (9)
C6	0.0619 (12)	0.0545 (11)	0.0690 (13)	0.0044 (9)	−0.0003 (10)	0.0015 (9)
N7	0.0731 (11)	0.0609 (11)	0.0687 (11)	0.0001 (9)	0.0042 (9)	−0.0105 (9)
O8	0.0977 (12)	0.0569 (9)	0.1100 (14)	0.0061 (9)	−0.0044 (10)	−0.0108 (9)
O9	0.0884 (12)	0.0789 (11)	0.0993 (13)	−0.0071 (9)	−0.0195 (10)	−0.0197 (10)
N10	0.0811 (12)	0.0523 (10)	0.0814 (13)	−0.0015 (8)	0.0031 (10)	0.0031 (9)
O11	0.0898 (12)	0.0568 (9)	0.1187 (15)	−0.0097 (8)	−0.0408 (11)	0.0116 (9)
C12	0.0720 (14)	0.0539 (11)	0.0716 (14)	−0.0052 (10)	−0.0151 (11)	−0.0015 (10)
C13	0.0787 (15)	0.0843 (16)	0.0693 (14)	0.0030 (13)	0.0001 (11)	−0.0023 (12)
C14	0.0888 (18)	0.0959 (19)	0.0906 (19)	0.0195 (15)	−0.0131 (15)	−0.0042 (16)
C15	0.119 (2)	0.0790 (17)	0.0801 (18)	0.0069 (17)	−0.0377 (17)	−0.0022 (15)
C16	0.148 (3)	0.088 (2)	0.0770 (18)	−0.031 (2)	0.0041 (19)	0.0114 (15)
C17	0.0778 (16)	0.0823 (17)	0.113 (2)	−0.0124 (13)	0.0075 (15)	−0.0026 (16)

### Geometric parameters (Å, °)

**Table:**

C1—C2	1.378 (3)	N10—H10A	0.8600
C1—C6	1.390 (3)	N10—H10B	0.8600
C1—N7	1.438 (3)	O11—C12	1.389 (3)
C2—C3	1.371 (3)	C12—C13	1.353 (4)
C2—H2	0.9300	C12—C17	1.367 (4)
C3—C4	1.385 (3)	C13—C14	1.359 (4)
C3—H3	0.9300	C13—H13	0.9300
C4—N10	1.355 (3)	C14—C15	1.337 (5)
C4—C5	1.412 (3)	C14—H14	0.9300
C5—C6	1.365 (3)	C15—C16	1.374 (5)
C5—O11	1.375 (3)	C15—H15	0.9300
C6—H6	0.9300	C16—C17	1.397 (4)
N7—O8	1.227 (2)	C16—H16	0.9300
N7—O9	1.227 (2)	C17—H17	0.9300
			
C2—C1—C6	121.29 (18)	C4—N10—H10B	120.0
C2—C1—N7	119.55 (18)	H10A—N10—H10B	120.0
C6—C1—N7	119.14 (18)	C5—O11—C12	120.32 (16)
C3—C2—C1	119.54 (19)	C13—C12—C17	121.1 (2)
C3—C2—H2	120.2	C13—C12—O11	117.8 (2)
C1—C2—H2	120.2	C17—C12—O11	121.0 (2)
C2—C3—C4	121.10 (18)	C12—C13—C14	120.2 (3)
C2—C3—H3	119.4	C12—C13—H13	119.9
C4—C3—H3	119.4	C14—C13—H13	119.9
N10—C4—C3	122.70 (19)	C15—C14—C13	120.9 (3)
N10—C4—C5	119.24 (19)	C15—C14—H14	119.6
C3—C4—C5	118.06 (18)	C13—C14—H14	119.6
C6—C5—O11	125.60 (19)	C14—C15—C16	119.9 (3)
C6—C5—C4	121.45 (19)	C14—C15—H15	120.1
O11—C5—C4	112.93 (18)	C16—C15—H15	120.1
C5—C6—C1	118.53 (19)	C15—C16—C17	120.1 (3)
C5—C6—H6	120.7	C15—C16—H16	119.9
C1—C6—H6	120.7	C17—C16—H16	119.9
O8—N7—O9	121.99 (19)	C12—C17—C16	117.9 (3)
O8—N7—C1	119.21 (17)	C12—C17—H17	121.1
O9—N7—C1	118.80 (18)	C16—C17—H17	121.1
C4—N10—H10A	120.0		

### Hydrogen-bond geometry (Å, °)

**Table:**

*D*—H···*A*	*D*—H	H···*A*	*D*···*A*	*D*—H···*A*
N10—H10A···O9^i^	0.86	2.17	3.023 (3)	170

Symmetry codes: (i) −*x*+1, *y*−1/2, −*z*+1/2.
